# Comparison of imaging changes in pulmonary artery diameter at the occlusion site using silk or metal clamps for pulmonary artery troubles

**DOI:** 10.1093/ejcts/ezae034

**Published:** 2024-02-09

**Authors:** Yoshiki Chiba, Masahiro Miyajima, Yuki Takahashi, Yuma Shindo, Kodai Tsuruta, Ryunosuke Maki, Atsushi Watanabe

**Affiliations:** Department of Thoracic Surgery, Sapporo Medical University School of Medicine and Hospital, Sapporo, Japan; Department of Thoracic Surgery, Sapporo Medical University School of Medicine and Hospital, Sapporo, Japan; Department of Thoracic Surgery, Sapporo Medical University School of Medicine and Hospital, Sapporo, Japan; Department of Thoracic Surgery, Sapporo Medical University School of Medicine and Hospital, Sapporo, Japan; Department of Thoracic Surgery, Sapporo Medical University School of Medicine and Hospital, Sapporo, Japan; Department of Thoracic Surgery, Sapporo Medical University School of Medicine and Hospital, Sapporo, Japan; Department of Thoracic Surgery, Sapporo Medical University School of Medicine and Hospital, Sapporo, Japan

**Keywords:** Anatomical lung resection, DeBakey type vascular clamp, Double-loop technique, Pulmonary artery troubles, Minimally invasive surgery, Video-assisted thoracoscopic surgery

## Abstract

**OBJECTIVES:**

We analysed our clinical experience using silk sutures [the double-loop technique (DLT)] or DeBakey type vascular clamp (DeBakey clamp) for pulmonary artery (PA) troubles during anatomical lung resection to validate its practicality and safety.

**METHODS:**

We retrospectively reviewed the records of patients who underwent either of the above clamping techniques during anatomical lung resection at our hospital between April 2007 and August 2022. We measured the PA diameter at the occlusion site on computed tomography images acquired within 1 year pre- and postoperatively. The difference between pre- and postoperative diameters of the occlusion sites was calculated as the change in the PA diameter. We zoned the occlusion site of the PA to adjust for variation. PA deformation was evaluated as an adverse event caused by clamping.

**RESULTS:**

Ultimately, 27 and 26 patients who underwent the DLT and DeBakey clamp, respectively, were included. No additional injury due to the clamp procedure was found in either group. For zone R1/L1, defined as the main PA, the median changes in the PA diameter were 0.02 (–0.7 to 0.27) mm for the DLT and 0.36 (–0.28 to 0.89) mm for the DeBakey clamp. No significant differences were observed between the 2 groups (*P *=* *0.106). Furthermore, no aneurysms, dissections, or stenoses were found in either group.

**CONCLUSIONS:**

The DLT and DeBakey clamp had only minimal effects on the occlusion site of the PA. The DLT is a practical thoracoscopic technique for PA bleeding when primary haemostasis has been achieved.

**Figure:**
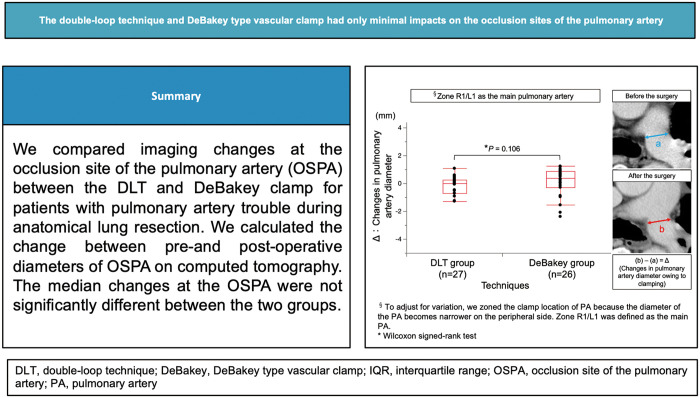

## INTRODUCTION

Traditionally, open conversion and the use of vascular clamp forceps to clamp the pulmonary artery (PA) have been used as the standard methods for cases of PA troubles during anatomical lung resection (ALR) [1, 2]. Recently, various clamp techniques available inside the thoracic cavity have been developed and used clinically along with the developments in minimally invasive surgery [3–5]. However, the currently used clamp techniques are only selected based on the surgeon’s experience. Therefore, we initiated a project to establish the objective evidence for the effectiveness and safety of each PA clamp technique, including the double-loop technique (DLT) developed at our institute, on PA. Initially, we reported an experimental study using an artificial vessel model that assumes the main PA to numerically compare the burst pressure and maximum clamp pressure of different clamp techniques [6]. Following this study, we analysed our clinical experience with the DLT. This study aimed to clinically validate the practicality and safety of the DLT and DeBakey type vascular clamp (DeBakey clamp) in cases of PA troubles.

## MATERIALS AND METHODS

### Ethics statement

The Sapporo Medical University Hospital Institutional Review Board approved this observational, retrospective, a single-centre study on 28 February 2023 (approval number: 342-210). The requirement for informed patient consent was waived owing to the retrospective nature of this study.

### Study design

Detailed patient data were collected from the electronic medical records, FileMaker Pro (Claris International Inc., Santa Clara, CA, USA) and surgical movies. The pathological stage of lung cancer was evaluated based on the eighth edition of the UICC TNM stage classification. Perioperatively, the following data were collected: patient characteristics, surgical approach, surgical procedure, operation time, bleeding amount, intraoperative trouble factor, clamp procedure, troubleshooting procedure and postoperative outcomes. Seven surgeons were included during the study period.

### Patients

We retrospectively analysed 1532 patients who underwent ALR for lung cancer, metastatic lung cancer or infectious lung disease at the Sapporo Medical University Hospital between April 2007 and August 2022. Patients who underwent DLT of the PA during video-assisted thoracoscopic surgery (VATS) were defined as the DLT group. Cases of DLT in open surgery were excluded because the DLT was defined as a clamp technique also available in VATS. In addition, we excluded patients without clamping and with division of the PA at the occlusion site, clamping with the bronchus and those with missing records. During the same period, we also analysed patients who underwent the DeBakey clamp because this was our usual clamp technique for PA troubles in cases requiring open surgery or total pulmonary arterioplasty. A few cases of Fogarty-type vascular clamp, the vessel loop technique and Satinsky-type partial vascular clamp were applied according to the surgeon’s preference, and these cases were excluded from our study.

### Surgical technique

The VATS approach with 1 window and 2 ports was used for thoracoscopic ALR. The patient was placed in the left or right lateral position. A mini-thoracotomy window (3–5 cm) was placed in the fourth intercostal space (ICS) for the upper lobe or the fifth ICS for the middle or lower lobes. Two ports (1.5 cm) were placed in the sixth or seventh ICS on the anterior axillary line, and in the seventh or eighth ICS on the posterior axillary line. Compression is the most common and basic method for haemostasis [2, 7]. We have used surrounding lung tissues or endoscopic instruments, such as a cotton dissector for compression, to control the bleeding. If haemostasis cannot be achieved with simple compression alone, fibrin sealant is used. Once primary haemostasis is achieved, the surgeon evaluates whether additional repair of the injured point is required after using the clamp technique.

The DLT was utilized thoracoscopically under the conditions of primary haemostasis achieved for PA injury. If the injured point is the peripheral side of the branch of the PA in the resected lung, the central side of the branch is ligated or stapled after clamping. Furthermore, the DLT was selected when the PA angiorrhapy was required. In our institute, angiorrhapy is performed using horizontal or Z suture with 4–0 or 5–0 polypropylene suture under the following circumstances: (i) the main PA or central side of the branch fissured without no bleeding and (ii) the ligature thread of the branch falls out. Whereas, we defined the circumferential PA arterioplasty as total pulmonary arterioplasty and the others as partial pulmonary arterioplasty. Partial and total pulmonary arterioplasties are performed using lateral running sutures with 4–0 or 5–0 polypropylene suture. DLT is indicated only if it is repairable with partial pulmonary arterioplasty. The DeBakey clamp becomes adaptive under the conditions of primary haemostasis not achieved for PA injury or total pulmonary arterioplasty required. During the open surgery, the DeBakey clamp was usually used in our department; however, other clamp techniques were rarely used according to the surgeon’s preference.

The criteria for open conversion at our hospital are as follows: in case of bleeding when primary haemostasis cannot be obtained; repeated re-bleeding; and when total arterioplasty of the PA is required. The extension of the operation time, degree of pleural adhesion and interlobar fusion were not considered indications for conversion.

The following criteria were used to select the occlusion site of the PA (OSPA): Upper lobe: (i) grand principle: more central side of main PA from the bleeding point; (ii) right: if the truncus superior (the first branch of the main PA) was processed, more peripheral side of the truncus superior; and (iii) left: main PA before branching out, regardless of whether the truncus superior was processed. Middle and lower lobes: more central side of main PA from the bleeding point. At the surgeon’s discretion, the peripheral side may be clamped or obstructed by compression with a cotton dissector. The criteria for intraoperative transfusion were as follows: abnormal vitals; haemoglobin <7.0 g/dl; or bleeding amount >1000 ml.

### Double-loop technique

The DLT is a thoracoscopic clamp technique using two 1–0 silk sutures. The procedure is demonstrated in detail in Video 1 and has been reported previously [3, 6]. The silk suture exerts a certain amount of frictional force on the surface; therefore, once the drawn loop is fixed, it is difficult to slip and loosen.

### Defining zone for the occlusion site of the pulmonary artery

**Figure 1:**
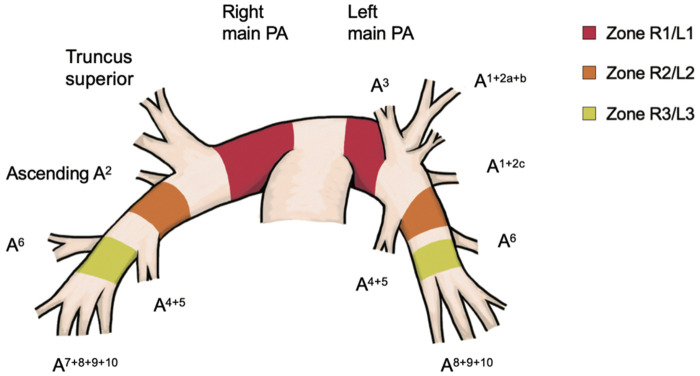
Zone for clamping to pulmonary artery. A: artery; PA: pulmonary artery.

To adjust for variation, we zoned the OSPA because the diameter of the PA becomes narrower on the peripheral side. The zone is defined as follows (Fig. 1):

Zone R1: Right main PA (more central than truncus superior).

Zone R2: PA (more peripheral than the ascending A^2^ branch and more central than the A^6^ branch).

Zone R3: Basal PA (more peripheral than the A^6^ branch).

Zone L1: Left main PA (more central than the A^1 + 2^ + A^3^ branches and more central if the lingual branch is mediastinal type).

Zone L2: PA (more peripheral than the A^1 + 2c^ and A^4 + 5^ branch and more central than the A^6^ branch).

Zone L3: Basal PA (more peripheral than the A^6^ branch and more peripheral if the lingual branch is interlobar type).

Clamping of the main PA included both inside and outside the pericardium.

### Perioperative outcomes

#### Trouble factors and troubleshooting

We classified surgical troubles related to the PA during VATS or open surgery as trouble factors. Ingrained lymph nodes were pathologically defined as metastatic or non-metastatic lymph nodes adherent to the PA. Furthermore, tumour invasion was pathologically confirmed to have the direct invasion of the tumour into the PA. Furthermore, we collected details of clamp procedures, such as the number of the OSPA, zone of the OSPA, clamp duration and additional injury due to the clamp procedure. Additionally, troubleshooting procedures for PA were also categorized.

#### Postoperative outcomes

Postoperative complications were evaluated according to the Clavien−Dindo classification and demonstrated to be over grade 1. Additionally, we also evaluated the duration of chest tube, postoperative hospital stay, postoperative blood transfusion, reoperation rate and 30- and 90-day mortality.

#### Postoperative adverse events on pulmonary artery

We retrospectively reviewed the surgical movies of enrolled patients and measured the diameter of the OSPA for the chest computed tomography (CT) images acquired within 1 year before and after the surgery. Additionally, the difference between the pre- and postoperative diameters of the OSPA was calculated as change in the PA diameter and was compared between the 2 groups (Supplementary Material, Fig. S1). To adjust for the conditions, only the central side was measured in cases in which central and peripheral clamps were performed. The presence of an aneurysm or stenosis as potential adverse events resulting from the clamp techniques used on the PA was evaluated. In addition, postoperative PA dissection was evaluated only in cases with enhanced CT scans.

### Statistical analysis

Continuous variables were expressed as median and interquartile range (IQR), and categorical variables were expressed as counts and percentages. To compare categorical variables between the DLT and DeBakey clamp, the Fischer’s exact test and chi-square test were used, and the Wilcoxon signed-rank test was used for continuous variables. Pre- to postoperative comparisons within the same group were made using the paired Wilcoxon signed-rank test. *P*-values 0.05 or less were considered significant. This study included 5 cases with missing CT images in our study. Because >10 years had passed since the surgery in all cases, the image data had been discarded. Therefore, these 5 cases could not be included in our study; however, all cases were confirmed alive at 1 year postoperatively. These 5 cases were classified as missing completely at random, therefore, we determined this study was acceptable to perform the complete case analysis that did not include these 5 cases [8]. Statistical analyses were performed using JMP Pro 15 (SAS Inc., Cary, NC, USA).

## RESULTS

**Figure 2:**
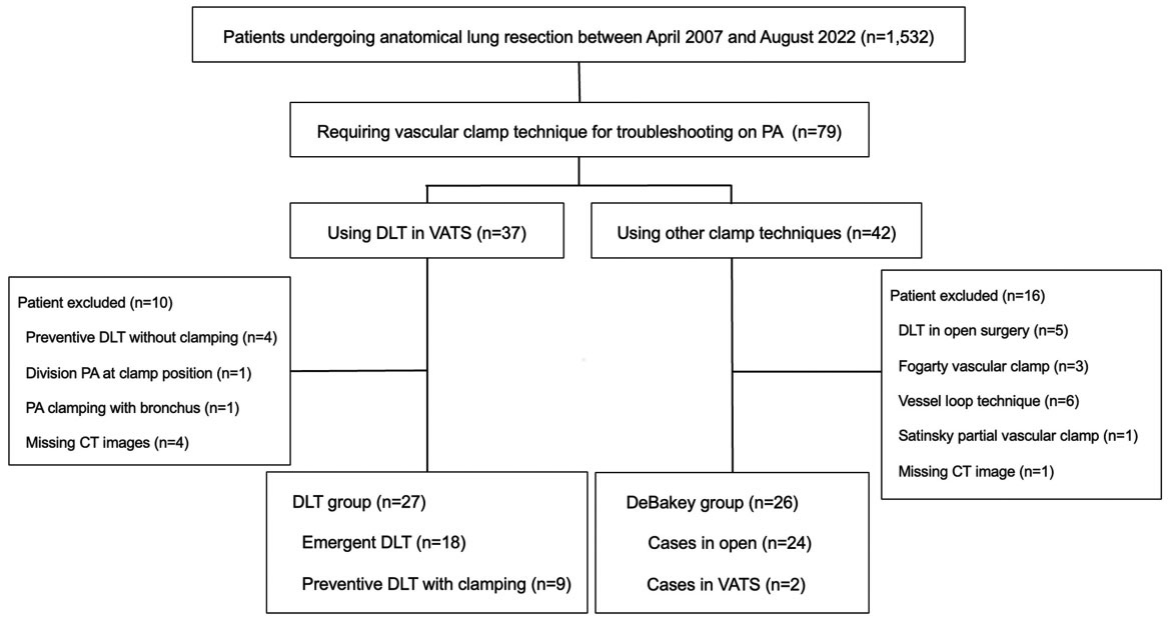
Patient flow chart. CT: computed tomography; DLT: double-loop technique; PA: pulmonary artery; VATS: video-assisted thoracoscopic surgery.

The patient flow chart is shown in Fig. 2. According to our selection criteria, 79 patients required clamp techniques for troubleshooting the PA. Finally, 27 patients were included in the DLT group. This group included patients who underwent emergent DLT (*n* = 18) for clamping owing to injury of the PA and patients who underwent preventive DLT (*n* = 9) for clamping owing to tumour invasion, an ingrained lymph node or ligature threads for the PA branch falling out. Furthermore, 42 patients underwent other clamp techniques, of whom 26 were included in the DeBakey group (open, *n* = 24; VATS, *n* = 2).

The patient characteristics are summarized in Table 1. Based on the histology of primary lung cancer, the DLT groups had more adenocarcinomas (*n* = 18, 66.7%), whereas the DeBakey group had more squamous cell carcinomas (*n* = 12, 46.2%, *P *=* *0.011). Additionally, the DeBakey group had a higher rate of stages IB–III (*n* = 19, 73.1%, *P *=* *0.035) compared to the DLT group. In the DLT group, 2 patients had stage IV cancer, 1 with N2 and another with rib metastasis. The DeBakey group had 1 patient who underwent neoadjuvant chemotherapy and another who underwent salvage ALR after chemoradiotherapy.

**Table 1:** Patient characteristics

Variables	DLT group (*n* = 27)	DeBakey group (*n* = 26)	*P*-value
Age (years), median (IQR)	68 (62–74)	68.5 (64–74)	0.810
Sex			0.254
Sex (male), *n* (%)	12 (44.4)	19 (73.1)	
Body mass index, median (IQR)	23.0 (20.8–25.5)	23.7 (20.4–25.9)	0.950
Comorbidities, *n* (%)			
Chronic obstructive pulmonary disease	9 (33.3)	8 (30.1)	1.000
Interstitial pneumonia	0	2 (7.7)	0.236
Cardiovascular disease	2 (7.4)	1 (3.9)	1.000
Pulmonary artery hypertension	0	0	–
Smoker, *n* (%)			0.327
Ever	19 (70.4)	22 (84.6)	
Never	8 (29.6)	4 (17.4)	
Brinkmann index, median (IQR)	400 (0–1100)	870 (475–1200)	0.137
FEV1.0 (l), median (IQR)	2.25 (1.73–2.70)	2.06 (1.43–2.50)	0.101
%VC, median (IQR)	117.8 (95.5–134.7)	92.6 (84.2–113.8)	0.008
Lesion size (mm), median (IQR)	23 (19–41)	33.5 (16–42)	0.306
Histology, *n* (%)			
Primary lung cancer			0.011
Adenocarcinoma	18 (66.7)	8 (30.8)	
Squamous cell carcinoma	3 (11.1)	12 (46.2)	
Adenosquamous carcinoma	1 (3.7)	0	
Neuroendocrine tumours	1 (3.7)	3 (11.5)	
Metastatic cancer to the lungs	3 (11.1)	3 (11.5)	1.000
Chronic progressive pulmonary aspergillosis	1 (3.7)	0	1.000
Pathological stage for primary lung cancer, *n* (%)			0.035
IA	10 (37)	3 (11.5)	
IB–IIB	4 (14.8)	8 (30.8)	
III	7 (25.9)	11 (42.3)	
IV	2 (7.4)	0	

DLT: double-loop technique; FEV1.0: forced expiratory volume; IQR: interquartile range; %VC: percentage of vital capacity.

The surgical procedures and outcomes are summarized in Table 2. In the DLT group, all cases were completed with VATS. The DLT group underwent more lobectomies than the DeBakey group, and 1 patient underwent completion left lingular division segmentectomy. In contrast, the DeBakey group had 13 patients (50%) who underwent VATS, of whom 11 (84.6%) required open conversion. Among them, 7 patients (53.8%) had emergent conversion because primary haemostasis could not be achieved. Patients in the DeBakey group underwent more plasty procedures than those in the DLT group. There were no significant differences between the groups in operation time or bleeding amount. However, patients in the DeBakey group underwent more intraoperative blood transfusions than those in the DLT group (*P *=* *0.050).

**Table 2:** Surgical procedures and outcomes

Variables	DLT group (*n* = 27)	DeBakey group (*n* = 26)	*P*-value
Approach, *n* (%)			<0.001
VATS	27	13 (50)	
Open	0	13 (50)	
Open conversion for VATS cases, *n* (%)	0	11 (84.6)	<0.001
Emergent	0	7 (53.8)	
Non-emergent	0	4 (30.8)	
Procedure, *n* (%)			
Lobectomy	19 (70.4)	9 (34.6)	0.014
RUL	4	2	
RML	1	0	
RLL	0	1	
LUL	11	5	
LLL	3	1	
Segmentectomy	8 (29.6)	4 (15.4)	0.327
RS^6^	0	1	
LUD	2	1	
LLD	1	0	
LS^1 + 2^	2	0	
LS^6^	2	0	
LS^6 + 10^	1	0	
LS^8 + 9^	0	1	
LS^8 + 9+10^	0	1	
Bi-lobectomy	0	3 (11.5)	0.111
Sleeve lobectomy	0	6 (23.1)	0.010
Double sleeve lobectomy	0	4 (15.4)	0.051
Operation time (min), median (IQR)	249 (206–299)	292 (239.8–403.3)	0.058
Bleeding amount (ml), median (IQR)	400 (150–650)	385 (173–1416)	0.327
Intraoperative blood transfusion, *n* (%)	1 (3.7)	6 (23.1)	0.050

DLT: double-loop technique; IQR: interquartile range; LLD: left lingular division; LLL: left lower lobe; LS: left segment; LUD: left upper division; LUL: left upper lobe; RLL: right lower lobe; RML: right middle lobe; RUL: right upper lobe; RS: right segment; VATS: video-assisted thoracoscopic surgery.

Trouble factors and troubleshooting procedures are presented in Table 3. The DLT group had a higher incidence of PA injury (*n* = 18, 66.7%) than the DeBakey group, of which ingrained lymph nodes were the most common reason (*n* = 10). The DeBakey group included more cases with difficulty in dissection (*n* = 13, 50%) compared to the DLT group, with tumour invasion to the PA being the most common reason (*n* = 11). In both groups, injuries to the PA were more common on the left side (DLT: *n* = 16, 59.3%, DeBakey: *n* = 8, 30.8%).

**Table 3:** Trouble factors and troubleshooting procedures

Variables	DLT group (*n* = 27)	DeBakey group (*n* = 26)	*P*-value
Trouble factors, *n* (%)			
Injury to the PA	18 (66.7)	12 (46.1)	0.035
Tumour invasion	0	2	
Ingrained lymph node	10	1	
Inflammatory adhesion	2	2	
Inadequate tissue handling	6	7	
Difficulty of dissection	7 (25.9)	13 (50)	<0.001
Tumour invasion	0	11	
Ingrained lymph node	7	2	
Falling out of ligature thread for the PA branch	2 (7.4)	0	1.000
Kink of the PA due to bronchoplasty	0	1 (3.9)	1.000
Injury point of the PA, *n* (%)			
Left	16 (59.3)	8 (30.8)	0.104
Main PA	1	3	
A^1 + 2^ + A^3^	6	1	
A^4 + 5^	3	1	
A^6^	4	0	
A^8 + 9+10^	2	3	
Right	2 (7.4)	4 (15.3)	0.392
Main PA	0	1	
A^1^ + A^2^ + A^3^	1	2	
A^4 + 5^	1	0	
A^6^	0	1	
Number of the OSPA, *n* (%)			0.054
One location	19 (70.4)	10 (38.5)	
Two locations	8 (29.6)	16 (61.5)	
Zone of the OSPA,^a^ *n* (%) (counted at only proximal location)			0.074
Zone R1/L1	19 (70.4)	23 (88.4)	
Zone R2/L2	7 (25.9)	1 (3.9)	
Zone R3/L3	1 (3.7)	2 (7.7)	
Clamp duration (min), median (IQR)	10 (8–20)	21.5 (10–58.5)	0.030
Additional injury due to clamp procedure	0	0	
Troubleshooting procedure for the PA, *n* (%)			
Plasty	10 (37)	16 (61.5)	<0.001
Partial	10	0	
Total	0	15	
Interposition	0	1	
Stapling	5 (18.5)	3 (11.5)	0.704
Angiorrhapy	11 (40.7)	6 (23.1)	0.241
Ligating and dividing by vessel sealing system	1 (3.7)	1 (3.9)	1.000

a The occlusion site is presented as a zone for the PA, which we defined in this study.

A: artery; DLT: double-loop technique; IQR: interquartile range; OSPA: the occlusion site of the pulmonary artery; PA: pulmonary artery.

Regarding clamping procedures, no difference in the number of cases in each zone of the OSPA was observed between the 2 groups. The median clamp duration (IQR) was 10 (8–20) min in the DLT group and 21.5 (10–58.5) min in the DeBakey group, with a longer duration in the DeBakey group (*P *=* *0.030). No additional injury due to the clamping procedure was found in either group. Furthermore, no patients were converted from the DLT to another clamp technique owing to the inability to achieve adequate occlusion. Regarding the troubleshooting procedure for PA, the DLT group underwent only partial pulmonary arterioplasty (*n* = 10, 37%). In the DeBakey group, total pulmonary arterioplasty was required in 15 patients, and interposition was required in 1 patient (*P *<* *0.001). In 9 out of 15 patients with total pulmonary arterioplasty and 1 patient with interposition, heparin (3–5 ml) was used during PA plasty at the surgeon’s discretion.

Postoperative outcomes are presented in Table 4. The DLT group had a shorter clamp duration and postoperative hospital stay than the DeBakey group did (*P *<* *0.001 for both). No postoperative haemorrhage occurred in either group. Recurrent nerve paralysis occurred in 3 patients (11.1%) in the DLT group and 2 patients (7.7%) in the DeBakey group; however, in all these patients, paralysis was due to the dissection procedure for the mediastinal lymph node, not the clamp procedure. Postoperative blood transfusion was required in 2 patients (7.4%) in the DLT group and in 5 patients (19.2%) in the DeBakey group (*P *=* *0.250). The DeBakey group had 1 patient who underwent bi-lobectomy caused by ipsilateral pulmonary embolism while on prophylactic anticoagulants. Then, the patient was managed by only oral anticoagulation. No reoperation or mortality occurred in either group. As for the PA deformation due to clamping, no aneurysm, dissection or stenosis was found in either group.

**Table 4:** Postoperative outcomes

Variables	DLT group (*n* = 27)	DeBakey group (*n* = 26)	*P*-value
Duration of chest tube (days), median (IQR)	2 (1–2)	3 (2–5)	<0.001
Postoperative stay (days), median (IQR)	11 (9–13)	17 (12.8–21)	<0.001
Postoperative complication, *n* (%)			0.867
Haemorrhage	0	0	
Anaemia	2 (7.4)	5 (19.2)	
Prolonged air leakage	3 (11.1)	3 (11,5)	
Pneumonia	2 (7.4)	2 (7.7)	
Atrial fibrillation	1 (3.7)	2 (7.7)	
Pulmonary embolism	0	1 (3.9)	
Recurrent nerve paralysis	3 (11.1)	2 (7.7)	
Vagus nerve paralysis	1 (3.7)	1 (3.9)	
Phrenic nerve paralysis	0	1 (3.9)	
Postoperative blood transfusion, *n* (%)	2 (7.4)	5 (19.2)	0.250
Reoperation	0	0	
30-Day mortality	0	0	
90-Day mortality	0	0	
Duration from operation day to first chest CT imaging after discharge (days), median (IQR)	114 (66–183)	120 (78.3–202.8)	0.669
Pulmonary artery deformation due to clamping			
Aneurysm	0	0	
Dissection^a^	0	0	
Stenosis	0	0	

a Postoperative pulmonary artery dissection was evaluated only in cases with enhanced CT imaging; 13 and 17 patients in the DLT and the DeBakey group, respectively.

CT: computed tomography; DLT: double-loop technique; IQR: interquartile range.

The median pre- and postoperative diameters (IQR) of PA were 18.1 (14.6–19.8) mm and 17.8 (14.9–19.5) mm in the DLT group and 19.6 (17.1–22.2) mm and 19.4 (17.3–22.3) mm in the DeBakey group, respectively. There were no significant differences between pre- and postoperative values in each group (*P *=* *0.857 and *P *=* *0.238).

**Figure 3:**
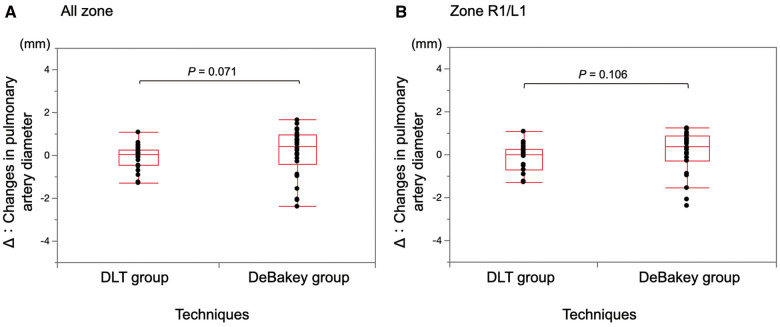
Comparison of the change in the pulmonary artery diameter at the occlusion site between the DLT and DeBakey clamp. (**A**) All zones. (**B**) Zone R1/L1. DLT: double-loop technique. Wilcoxon signed-rank test.

A comparison of the change in the PA diameter at the OSPA between the DLT and DeBakey clamp is presented in Fig. 3A and B. For all zones, the median changes in the PA diameter (IQR) were 0.03 (−0.46 to 0.27) mm in the DLT group and 0.44 (−0.43 to 0.96) mm in the DeBakey group. For zone R1/L1, the median changes in the PA diameter (IQR) were 0.02 (−0.7 to 0.27) mm in the DLT group and 0.36 (−0.28 to 0.89) mm in the DeBakey group. Additionally, for all zones and zone R1/L1, no significant differences were observed between the 2 groups (*P *=* *0.071 and *P *=* *0.106).

## DISCUSSION

To the best of our knowledge, this is the first study to evaluate the postoperative effects of clamping of the PA, including the main PA, defined as zone R1/L1. Previously, we demonstrated that the DLT had a pressure resistance capacity and intimal load as similar to the DeBakey clamp at the third notch [6]. Consequently, this clinical study showed that the DLT itself does not cause additional injury to the PA during ALR or postoperative adverse events on the OSPA. No postoperative deformations of the clamped PA were observed in either group. Additionally, the DLT group was comparable to the DeBakey group regarding the change in diameter at the OSPA for all zones. We believe that our research established clinical evidence that both the DLT and DeBakey clamp, which had been used almost empirically, are feasible and safe clamp techniques. Furthermore, the DLT does not require an extended incision or an additional incision for clamping, can be completed intrapleurally and allows the surgeon to control the clamp pressure by gradually drawing the encircling silk [3, 6]. Additionally, preventive DLT was clinically performed in 13 patients (including 4 patients using preventive DLT without clamping). This technique can easily be introduced during ALR with VATS if the surgeon has the skill to dissect and expose the main PA. Our clinical study underscores the advantages of the DLT, which can be performed under appropriate conditions during ALR with VATS.

In the DeBakey group, squamous cell carcinoma, central lesions and advanced stages of lung cancer were common. Furthermore, patients in the DeBakey group had a longer clamp duration, duration of chest tube and postoperative hospital stay than those in the DLT group. These differences could be because the DeBakey clamp was used in more advanced cases in deference to the selection criteria for the DLT or DeBakey clamp. For the same reason, the DeBakey group had a longer clamp duration than the DLT group did. Nevertheless, there was no difference in the change in the diameter between the 2 groups. The relationship between clamp duration and intimal load on the PA is unclear. Although the DeBakey clamp can reliably obstruct the PA, it interferes with the thoracoscopic view and generally requires extending the incision or placing an additional incision for safe use. The notch of the vascular clamp forceps also defines the clamp pressure; however, only changing 1 more notch significantly increases the clamp pressure [6]. Therefore, vascular clamp forceps cannot adjust the clamp pressure delicately. Our previous study showed that the DeBakey clamp at the fourth notch might overload the PA intima [6]. This result demonstrated that the surgeons should always carefully select the notch of the vascular clamp forceps. Contrary to the results of our experimental study, our clinical study found no adverse events in clamped PA. Based on these results, we hypothesized that the low-pressure system of the PA is unlikely to undergo deformation despite the potential intimal damage to the clamped PA.

### Limitations

The study had certain limitations. The sample size was small because we encountered few cases requiring clamp techniques during ALR in our single centre. Furthermore, the baseline variables were not well balanced between the 2 groups because of differences in the selection criteria for the DLT or DeBakey clamp. The groups were not precisely comparable. However, as in the background for this clinical study, the histological analysis of the effectiveness and safety of each clamp technique for the human main PA is quite difficult [6]. Therefore, we believe that this clinical study was worthwhile because of the inclusion of the main PA as zone R1/L1. Enhanced CT was not performed in all cases, and the presence of a PA dissection was only assessed in about half of the cases in both groups. Furthermore, this long-term retrospective study differed in CT equipment and imaging conditions. In this study, 7 surgeons performed the operation, and there was no established rule for choosing the clamp technique.

## CONCLUSION

This study using CT images demonstrated that both the DLT and DeBakey clamp had only minimal impacts on the OSPA. The DLT is a practical thoracoscopic technique for PA bleeding if primary haemostasis has been achieved. A further histological study is underway to reach a consensus on its safety.

## Supplementary Material

ezae034_Supplementary_Data

## Data Availability

Data will be shared with the authors upon request.

## Notes

Presented at the 40th Annual Meeting of the Japanese Association for Chest Surgery, Niigata, Japan, 14 September 2023.

## ABBREVIATIONS

ALR: Anatomical lung resection
CT: Computed tomography
DeBakey clamp: DeBakey type vascular clamp
DLT: Double-loop technique
IQR: Interquartile range
OSPA: Occlusion site of the pulmonary artery
PA: Pulmonary artery
VATS: Video-assisted thoracoscopic surgery
