# Supplementary figures and images for: Comparison of imaging changes in pulmonary artery diameter at the occlusion site using silk or metal clamps for pulmonary artery troubles
Source: Eur J Cardiothorac Surg. 2024 Feb 9;65(3):ezae034. doi: 10.1093/ejcts/ezae034 (PMC11024802; doi:10.1093/ejcts/ezae034)

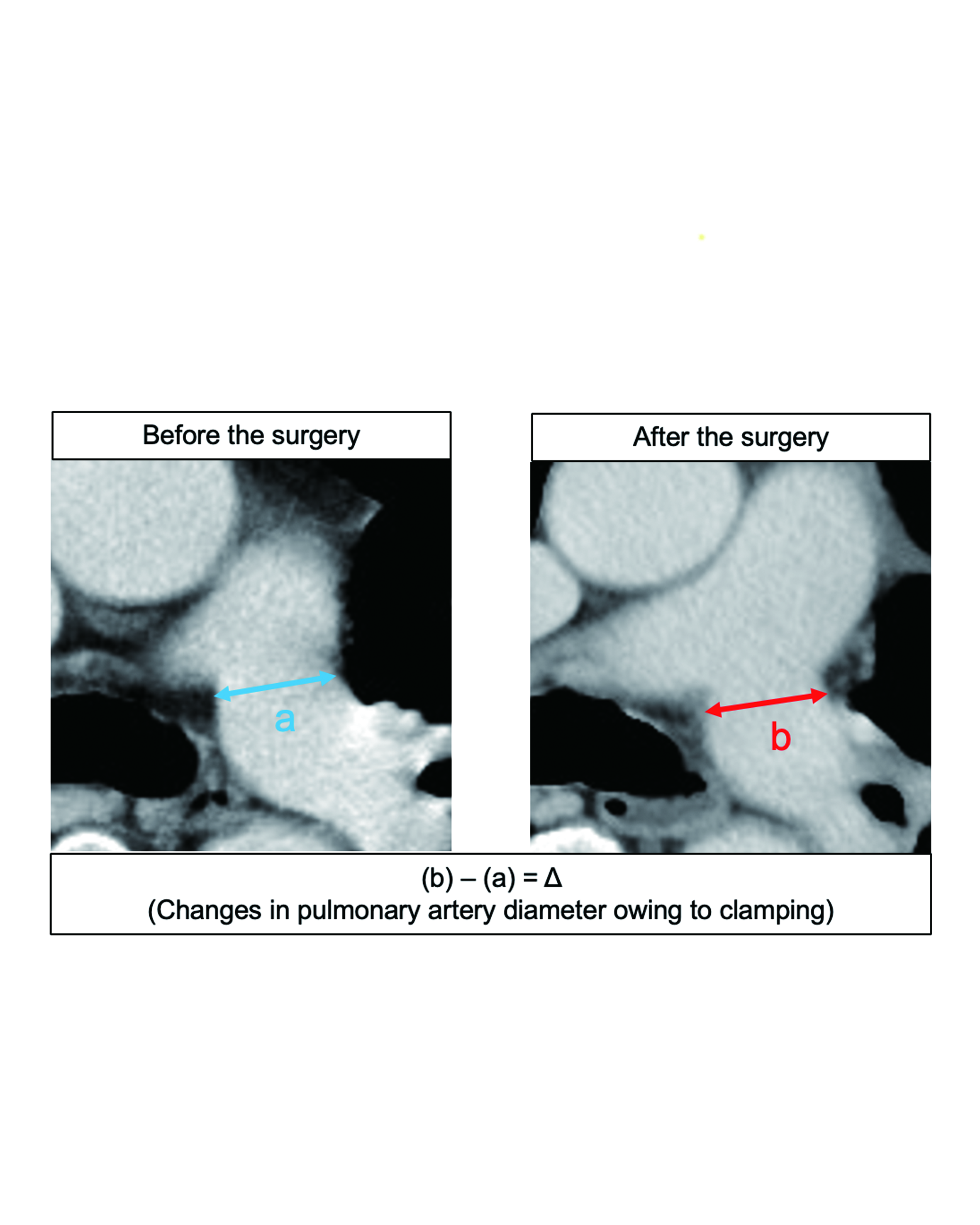

Supplement: ezae034_Supplementary_Data [file ezae034_Supplementary_Data.tif]
